# The Zebrafish Xenograft Models for Investigating Cancer and Cancer Therapeutics

**DOI:** 10.3390/biology10040252

**Published:** 2021-03-24

**Authors:** John T. Gamble, Daniel J. Elson, Juliet A. Greenwood, Robyn L. Tanguay, Siva K. Kolluri

**Affiliations:** 1 Department of Biochemistry & Biophysics, Oregon State University, Corvallis, OR 97331, USA; gamble.john@epa.gov; 2 Cancer Research Laboratory, Department of Environmental and Molecular Toxicology, Oregon State University, Corvallis, OR 97331, USA; elsond@oregonstate.edu; 3 School of Mathematics and Natural Sciences, Arizona State University, Scotsdale, AZ 85257, USA; juliet.greenwood@asu.edu; 4 Department of Environmental and Molecular Toxicology, Oregon State University, Corvallis, OR 97331, USA; robyn.tanguay@oregonstate.edu

**Keywords:** xenograft, zebrafish, cancer therapeutics, drug discovery, drug screening, toxicity testing

## Abstract

**Simple Summary:**

The identification and development of new anti-cancer drugs requires extensive testing in animal models to establish safety and efficacy of drug candidates. The transplantation of human tumor tissue into mouse (tumor xenografts) is commonly used to study cancer progression and to test potential drugs for their anti-cancer activity. Mouse models do not afford the ability to test a large number of drug candidates quickly as it takes several weeks to conduct these experiments. In contrast, tumor xenograft studies in zebrafish provide an efficient platform for rapid testing of safety and efficacy in less than two weeks.

**Abstract:**

In order to develop new cancer therapeutics, rapid, reliable, and relevant biological models are required to screen and validate drug candidates for both efficacy and safety. In recent years, the zebrafish (*Danio rerio*) has emerged as an excellent model organism suited for these goals. Larval fish or immunocompromised adult fish are used to engraft human cancer cells and serve as a platform for screening potential drug candidates. With zebrafish sharing ~80% of disease-related orthologous genes with humans, they provide a low cost, high-throughput alternative to mouse xenografts that is relevant to human biology. In this review, we provide background on the methods and utility of zebrafish xenograft models in cancer research.

## 1. Zebrafish as Model for Studying Human Cancer

Cancer drug discovery requires both in vitro screening and validation in human cell models and in vivo testing in animal models. Cell models provide the ability to screen numerous potential therapeutics rapidly against many different cancer types. Traditionally, genetically engineered mouse models or xenografts of human cancer cells using immunocompromised mice (e.g., NOD-SCID) have been considered the gold-standard for testing both safety and anticancer drug efficacy in vivo. Experiments utilizing mice require relatively longer time and are generally an unsuitable platform for high-throughput screening and toxicity testing of anticancer drugs.

Zebrafish have become an established tool for both drug discovery and evaluating toxicity [1,2]. Zebrafish possess numerous characteristics that make them an attractive model for cancer drug development. In contrast to mice, they are highly fecund and display rapid development. Mating can yield hundreds of fertilized eggs that rapidly develop from embryos into larval fish upon hatching at approximately 72 h post-fertilization (hpf) [3]. At this stage they possess core features of vertebrates, namely: notochord, pharyngeal arches, posterior tale, and somites [3]. Zebrafish reach breeding maturity by 90 days post-fertilization. Although they are not mammals, zebrafish have a comparable vertebrate anatomy to humans and have orthologs for 70% of human proteins [4]. With current technology, it is relatively easy to generate zebrafish transgenic models with altered expression of genes. The use of transgenic zebrafish cancer models has enabled the study of numerous malignancies [5,6], and transgenic models with vascular endothelial cell-specific expression of fluorescent proteins have enabled discoveries related to developmental and tumor-induced neo-angiogenesis and vascularization [7]. Morpholino technology has become a niche tool of choice in the zebrafish field for modifying gene expression [8,9]. Morpholino oligonucleotides can be injected into fertilized single cell eggs to silence complementary mRNA and suppress specific protein translation in early zebrafish development [9]. While the utility of morpholinos as a tool for modifying gene expression in zebrafish has been phenomenal, morpholinos sometimes produce off-target effects and morpholino-induced phenotypes can be more severe than that of the corresponding genetic knockout mutant. In order to address these issues, guidelines and best practices have been outlined to validate observations derived from morpholino experiments in zebrafish [9,10]. These attributes provide valuable tools with the flexibility to answer many different cancer biology questions, including cancer drug discovery.

## 2. Embryo-Larval Zebrafish Xenografts

**Figure 1:**
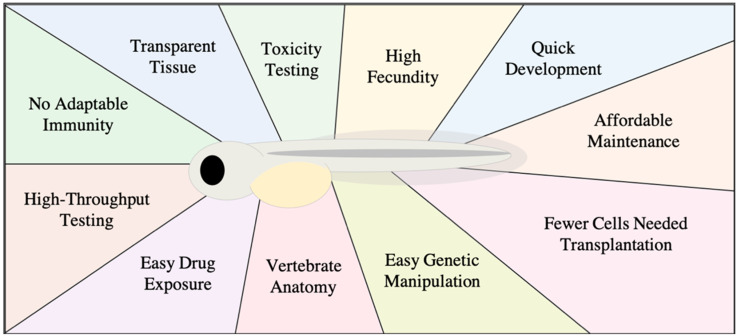
Diagram of advantages for using a zebrafish xenograft model.

Xenograft transplantation is the relocation of living cells from one species to another. Zebrafish are an advantageous model for human cancer cell transplantation [11]. The transplanted human cells not only survive but are able to migrate and interact with the host environment. Zebrafish xenograft experiments have successfully enabled the study of various human cancer lines, such as those from melanoma, breast, and leukemia, among numerous other cancer types [12,13,14,15]. The embryo-larva zebrafish offers a number of advantages over other xenograft models (Figure 1). Zebrafish possess innate immune cells but lack an adaptive immune system in early life, developing a functional adaptive immune system only after 30 days post-fertilization [16]. This attractive characteristic permits xenotransplantation of human cancer cells without immune rejection, and precludes the need for immunosuppressive drugs or immunocompromised variants to perform xenografts. Zebrafish are millimeters in size and therefore can be kept in petri dishes together or individually in 96-well plates, enabling facile handling and maintenance. Despite zebrafish preferring an environmental temperature of 28 °C, they are capable of surviving at temperatures from 32 to 36 °C, closer to human cell culture conditions [17,18,19]. Additionally, zebrafish transplantation requires only hundreds of cells or fewer, while mouse xenografts require several times as many [19]. This is of particular importance when cancer cell numbers are finite, such as those derived from primary patient tissue samples. Furthermore, within approximately two days post-fertilization, zebrafish embryos provide a complement of orthotopic organs and tissues such as the brain, heart, and liver as well as a functioning circulatory system [3,20].

Zebrafish offer accessibility for in vivo testing as they require relatively minimal maintenance and care relative to mammalian models. Moreover, zebrafish provide a model where cancer progression can be assessed by imaging equipment available to most researchers, such as standard epifluorescence and confocal microscopes. Due to the transparent nature of embryo-larva zebrafish, assessment of potential drug effects can be evaluated non-invasively within the host. Transparent zebrafish tissue provides extraordinary optical penetration allowing collection of detailed images of fluorescent cancer cells. With the organism’s tissue thickness in the micron scale, excitation/emission spectrums of fluorescent probes escape the tissue with little light scattering, particularly at longer wavelengths. Fluorescent proteins such as mCherry and green fluorescent protein (GFP) and fluorescent dyes such as CM-DiI and 5-chloromethylfluorescein diacetate (CMFDA) have been successfully used to not only visualize individual cells in zebrafish, but also subcellular structures, i.e., centrosomes, endosomes, mitochondria, microtubules, etc. [21,22,23,24]. Different labeling approaches carry respective advantages and drawbacks. Lipophilic fluorescent dyes, for example, are easy to utilize, but can have effects on cell viability at higher concentrations. In contrast, expression of fluorescent proteins can be time-consuming to generate. The relative advantages and disadvantages of different labeling strategies have been thoroughly discussed elsewhere [25].

**Figure 2:**
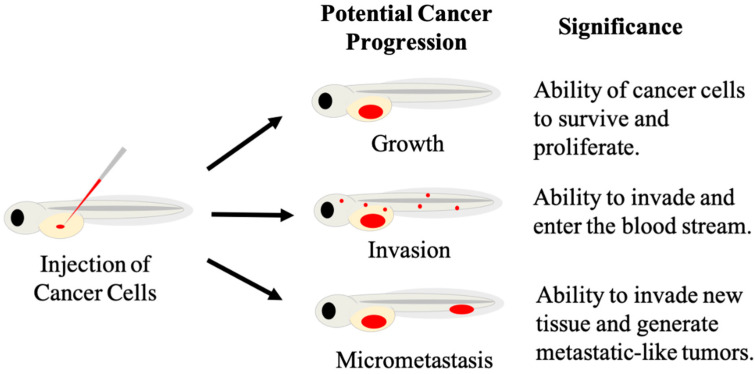
Diagram depicting measurable signs of cancer progression in zebrafish xenografts. Human fluorescent cancer cells (red) are injected into the yolk of embryonic zebrafish (left), with injected cells represented by the red oval. Cancer progression can display as growth, invasion or micrometastases (center). These phenotypic responses can be measured to provide information on specific cancer behavior, and responses of cancer cells to different treatments.

The first published zebrafish xenograft utilized the embryonic blastodisc region for injection at 3.5–4.5 h post-fertilization, and followed the fate of injected melanoma, and noncancerous human fibroblasts and melanocyte [11], since then, numerous other injection sites have been adopted and exploited for xenografts. Beyond the yolk sac, injection sites include the duct of cuvier, also known as the common cardinal vein, where injection into the bloodstream is used to study the process of metastasis. In addition, the caudal vein, perivitelline space, and hindbrain ventricle of the brain have also been used for xenografts [26]. The zebrafish yolk provides an in vivo environment for human cancer cells to proliferate and migrate [26]. At the embryonic and larval stages, zebrafish rely on their yolk to supply them with the necessary nutrients to grow and develop. The yolk provides a nutrient rich environment hospitable to engrafting hundreds of cancer cells that is rich in cholesterol, lipids, and phosphatidylcholine. The yolk sac’s size aids in the transplantation process as it provides a relatively large injection site that is easy to identify and transplant into with a microinjector. The most commonly used timepoint for yolk sac injections is 48 hpf, and this method was first established by Haldi, et al. [12]. The rationale for injection at 48 hpf stems from the large size of the yolk sac at this stage, and the completion of gastrulation, thus injected cancer cells are not expected to passively diffuse throughout the developing organism. Additionally, the establishment of vasculature and rudimentary organ systems reduces the risk of severe developmental defects incurred by the injection procedure. Many different human cancer cell lines, including breast [27], neuroblastoma [28], melanoma [11,12,29], leukemia [14,15,30], prostate [31], and ovarian cancers [32], among numerous others [26] can survive and proliferate in the zebrafish yolk. With transplantation into the yolk, many different cancer cell characteristics can be assessed with the most predominant endpoints being growth, survival, invasion, and metastasis (Figure 2). Cancer cell proliferation can be prolific as malignant cells can quadruple in three days while inside the zebrafish yolk [12]. Aggressive cancer cells can quickly migrate and exit the yolk sac by entering the blood stream to travel throughout the body [33]. Transplanted patient-derived gastrointestinal tumor cells form micrometastases in zebrafish unlike non-tumorigenic primary cells [13]. Moreover, zebrafish xenograft experiments have replicated results of experiments conducted in mouse xenografts. Breast, prostate, colon, and pancreatic cancer cell lines that metastasized in mice also metastasized in zebrafish, while non-metastasizing cell lines did not [34].

Blood vessels are an integral part of tumor development. Zebrafish have been used to measure tumor cell and blood vessel interactions. To stimulate blood vessel growth, tumor cells secrete angiogenetic factors [35]. To study tumor-induced angiogenesis, zebrafish with vascular endothelial cell-specific expression of GFP have been generated [7,12,36,37]. Two of the most commonly used GFP-expressing zebrafish lines leveraged for studying developmental and tumor-induced angiogenesis include the *fli1a:EGFP* [38] and *kdrl:EGFP* [39] lines. Within 24 h of implantation, angiogenesis can be measured as zebrafish blood vessels are seen growing towards transplanted human tumor cell masses [12,40,41]. Zebrafish blood vessels offer the necessary architecture to assess metastatic characteristics, like extravasion and intravasion, of tumor cells. With metastatic cancers, cancer cells must enter, intravasion, into the blood stream and exit, extravasion, back out in order to travel to other organs and tissues [42]. Tumor cells transplanted into the blood stream of zebrafish are capable of extravasion as tumor cells can be seen attaching to the endothelium and exiting the capillaries [43]. Intravasion can also be seen, as tumor cells transplanted into the yolk can invade into the blood stream, travel to the tailfin and form micrometastases [34]. The use of zebrafish has led to important insights into the mechanisms of sprouting angiogenesis [44,45,46,47], vessel guidance [41,48], and vascular endothelial growth factor signaling [49,50], among other angiogenic processes [51].

Although zebrafish lack certain organs present in humans (i.e., lungs, mammary pads), orthotopic transplantation is possible for many cancers, with the brain being the most widely used. The brain is developed with fore-, mid- and hindbrain ventricles by the end of the zebrafish embryonic stage [3]. These ventricles provide a pocket for cancer cells to occupy. The larval zebrafish brain provides a complex environment with glia, neurons, and blood vessels held together by a unique extracellular matrix ECM [52,53,54]. A functioning vasculature permeates the brain as well, where pericytes and endothelial cells provide a basement membrane and protective blood brain barrier by 4-day post fertilization [55]. Transplanted human glioma cells in a zebrafish brain environment exhibit behavior seen in human patients as cells aggressively invade the surrounding tissue while migrating and along cranial blood vessels [19]. Furthermore, to maneuver through the environment, glioblastoma cells can be seen transitioning between amoeboid and mesenchymal type mechanisms to squeeze and pull through the tight spaces in the brain [52,56].

Time-lapse imaging can provide insights into cancer cell behavior and shed light on mechanisms necessary for migration, invasion, proliferation, and metastasis. Zebrafish xenografts provide visual accessibility to an in vivo tumor-like environment. Using zebrafish xenografts, it was possible to track individual glioblastoma cell movement throughout the brain [19,57,58,59].

## 3. Zebrafish Xenografts for Cancer Drug Screening

While drugs may be effective and efficient at killing or hindering cancer growth in vitro, they might cause complex problems in multicellular systems. Rodent models are the standard for drug toxicity testing, but it is not practical to conduct detailed tests on hundreds of compounds for early-stage discovery. In an effort to bridge this gap, the embryo-larva zebrafish model is an established, sensitive and predictive model for human toxicity. Humans and zebrafish share similarities in embryogenesis, with conserved gene expression across phyla, as well as many shared anatomical and physiological structures and functions [2,60]. Systemic toxicity can be quickly assessed as treatments are added directly to the fish media. Easily observable toxic effects may include reduced survival or phenotypic abnormalities, and additional developmental and behavioral endpoints can be monitored non-invasively [61]. Beyond overt, morphologically obvious toxic phenotypes, additional tools have been developed for monitoring drug toxicity in zebrafish [1,62]. One of the most frequent off-target toxicities of drugs occurs against blood cells [63]; correspondingly a transgenic zebrafish model has been generated for monitoring potential hematotoxicity, utilizing an erythroid-specific fluorescent reporter to measure changes in blood cell number following drug treatments [62]. Toxicity against the nervous system can be monitored using numerous assays, including the photomotor response assay, which is widely employed for detecting behavioral effects [1].

The embryo-larva zebrafish’s diminutive size is conducive to cancer drug screening. With their small size, single or multiple zebrafish can be stored in 96-well plates during the embryo-larval stage. The 96-well format confers the ability to treat quickly and easily with multiple concentrations of different small molecules [64]. Furthermore, each embryo-larva requires micro-liter volumes of media and therefore treatments are accomplished with minimal quantities of test chemicals. Moreover, images of the entire fish can be captured with a wide-field objective allowing for rapid acquisition of phenotypic effects. Using high-content microscopes, this process offers the ability to collect data on large numbers of xenografts [65,66].

**Figure 3:**
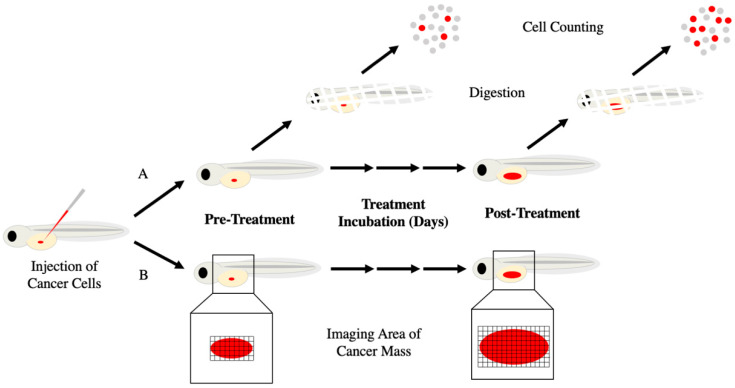
Methods of calculating cancer growth in zebrafish xenografts. Zebrafish embryos are engrafted with fluorescent cancer cells (red) by microinjection into the yolk sac and tumor progression is assessed in one of two ways. (**A**) Pre-treatment, a random subset of zebrafish is sacrificed, digested with collagenases, and fluorescent cells are counted. The remainder of the xenografts are exposed to treatment. After the treatment period, xenografts are digested, and cells are counted as before. Fold change in cell count reflects cancer growth/reduction. (**B**) Images are taken of the fluorescent cancer cells within the zebrafish at both pre- and post-treatment with fold change in area and fluorescent intensity indicating cancer growth/reduction.

There are different methods to assess changes in cancer growth with zebrafish xenografts (Figure 3). All methods start with transplantation of fluorescent cancer cells into embryonic zebrafish and allowing the animal to continue to develop and grow over several days. Some methods require sets of zebrafish be euthanized before and after treatment and enzymatically dissolved to produce a unicellular suspension. Fluorescent cells are then fixed and counted on a hemocytometer or flow cytometry to compare between treatment groups [12,30]. Collection and counting cells from euthanized zebrafish provide a more accurate proliferation rate of the cancer cells. Additionally, with other staining procedures, live and dead cells can be counted as well as protein expression levels using fluorescent antibodies. However, due to euthanasia there can no longer be a time course comparison for each animal and therefore less statistically powerful unpaired group comparisons must be relied upon [67]. This is of importance as each zebrafish xenograft may have a different number of transplanted cells due to the nature of the micro injections. Because zebrafish xenograft transplantations engraft a small population of cells, slight variations in transplantation numbers can result in large percentage differences between xenografts. Furthermore, this method is labor intensive and therefore limits the capability for screening of many potential drugs. Alternatively, images of the fluorescent cells in the zebrafish are taken before and after treatment without euthanizing the host [18,19,57,58,68,69]. Fluorescent cell area and intensity are used to measure cancer growth. By imaging the cells within individual zebrafish, direct comparisons can be made within and between animals over time. This results in a more powerful statistical paired test and higher N value. Additionally, high-content imaging microscopes are used to help automate the process, giving their capability for high-throughput. However, cells cannot be counted individually as they are typically indistinguishable in a large mass. Changes in area and intensity are relied upon for growth information and because increases in both can be correlated to cancer growth.

Patient-derived xenografts (PDX) provide a more authentic view to investigate cancer. Like mouse models, zebrafish can be a host for patient tumor cells and tissues. Indeed, PDXs utilizing zebrafish as “avatars” for rapidly evaluating potential therapies are emerging as exciting tools [70,71]. Zebrafish patient-derived xenografts have been shown to display similar cancer behavior to that seen in the cancer patient. For instance, transplanted bone metastasis tumor cells from a patient with breast cancer were seen migrating to caudal hematopoietic tissues, comparable to human bone morrow, in zebrafish xenograft [72]. Transplantation of whole patient tissue was also demonstrated with pancreatic tumor where it was implanted into the yolk of embryonic zebrafish and displayed similar metastatic behavior to that seen in the patient [14]. In order to select proper drug treatment for an individual, biopsy samples can be processed and transplanted into zebrafish in the same manner as described here. As zebrafish embryo-larva require a minimal number of cancer cells, many more xenografts can be generated from a single patient than traditional murine models. With numerous xenografts, many different treatments can be tested with results produced within days. Patient biopsies were transplanted into zebrafish and tested for responsiveness to the anti-angiogenic drug bevacizumab in a proof-of-concept study for zebrafish avatars [73]. Tumor responsiveness, the impact on angiogenesis, and number of micrometastases in zebrafish, were measured before administering drugs to the patients, and comparing their respective responses. Zebrafish PDX models have the potential to provide medical providers with personalized experimental data on drugs for deciding an effective individualized treatment regimen. The ability to generate this personalized data rapidly has the potential to greatly extend patient lifespan as patients with aggressive cancers need appropriate effective treatment quickly.

To our knowledge, no FDA-Approved Drugs have been approved to date based on leads discovered in zebrafish xenograft models. However, zebrafish xenograft metastatic models have established the basis for at least two newly identified pathways for pharmacological intervention: a YAP1-CXCR2 dependency [74,75], and PIT1-CXCR4 [27,76] signaling axis of which there are cognate targeted compounds currently under clinical investigation [77]. Using a yolk-sac metastasis model, the transcriptional coactivator Yes-associated protein (YAP) was shown to drive increased expression of the cytokines interleukin-6, interleukin-8, and C-X-C motif ligands 1, 2, and 3, that in turn promoted cancer cell vascular invasion [75]. Separately, secreted interleukin-8 binding to the G-protein coupled, chemokine CXCR2 was also shown to drive metastasis in zebrafish, and small-molecule inhibition of CXCR2 abolished paracrine invasion [74]. Like CXCR2, the chemokine receptor CXCR4 and its cognate ligand CXCL12 have been established as important mediators of migration and invasion. It was demonstrated that xenografted human triple-negative breast cancer cells could cross-communicate with and respond to the zebrafish CXCL12 ligand, and that chemical and genetic disruption of this axis potently reduced invasion [76]. The transcription factor POU1F1 (also known as Pit-1) was demonstrated to be an important driver of CXCR4 and CXCL12 expression and contributes to pro-angiogenic signaling and invasiveness in vivo [27].

In non-metastatic models, several compounds that demonstrated efficacy in zebrafish xenografts have entered clinical trials, including the c-MET inhibitor crizotinib for uveal melanoma [78,79], and BPIQ—an irinotecan analog for non-small cell lung cancer [80]. It is expected that more drug targets and leads will be identified using zebrafish xenografts in the future.

### 3.1. Adult Zebrafish Xenograft Models

With adult zebrafish possessing a fully developed immune system relative to their larval stages, xenograft studies in adult fish have relied on immunosuppressive techniques to blunt graft rejection, including dexamethasone treatment [43] and gamma irradiation [81]. Adult zebrafish model systems provide several advantages relative to larval xenograft studies. Larval xenograft models are confined to <10 days due to the development of an adaptive immune system after this period, while different immunodeficient adult fish models can be monitored for a much longer time span. Recently, the generation of genetic models of immunodeficient adult zebrafish [82,83,84] have circumvented these interventions, by breeding fish that lack [84], or possess only partial [82,83] adaptive immune responses. Immunosuppressed adult zebrafish models were developed by the introduction of mutations into either recombination activating gene 2 (*rag2*), protein kinase DNA-activated catalytic polypeptide (*prkdc*), or janus kinase 3 (*jak3*) leading to different deficiencies in the generation of, or maturation of B cells, T-cells, and natural killer (NK) cells in adult zebrafish possessing a Casper genetic background [82]. Following characterization of these mutant lines, the optically clear *prkdc*-mutant zebrafish (lacking mature T and B cells) was used as a platform to demonstrate the engraftment of normal and malignant human cells into mature zebrafish [82]. Pioneering work subsequently led to the generation of an improved immunodeficient adult zebrafish model [84], where deficiency of *pkrdc* and *il2rga* (interleukin-2 receptor gamma a) lead to fish deficient in B, T, and NK cell production and capable of highly efficient engraftment of human cancer cells. Notably, the prkdc^fb103/fb103^ il2rga^fb104/fb104^ Casper-strain zebrafish model is capable of being reared at 37 °C, compared with larval fish models that require rearing at 34 °C, and thus provide a more representative physiological model for predicting human tumor responses. Due to the larger body size of adult fish, a greater number of cancer cells can be engrafted, increasing the likelihood of capturing more rare cell populations, such as cancer stem cells. Adult fish can receive drug/compound treatments by oral gavage, compared with immersion dosing for larval fish, and pharmacokinetics can be monitored. Importantly, comparison of histology, proliferation, and apoptosis rates between adult zebrafish and mouse xenografts shows that engrafted cancer cells, of diverse origin, behave similarly between the two models [84]. Finally, the transparent nature, and ability to monitor adult fish over a longer time (>28 days) enables new methods for studying cancer cell dynamics, such as extended profiling of single-cell behavior, discussed in the next section.

### 3.2. New Extended Utility of the Zebrafish Xenograft Model

Despite a high degree of protein sequence homology between humans and zebrafish, zebrafish express significantly different, or lack altogether, certain cytokines and receptors required for the differentiation and maintenance of select cell types, including factors necessary for hematopoietic stem cell and progenitor (HSPC) clonal proliferation and survival [85]. While human leukemia xenografts have been successfully achieved in zebrafish models [14,30], maintenance of the HSPC niche, and durability of HSPC clonal expansion post-injection has been a subject of uncertainty [15,86]. Excitingly, a humanized zebrafish model was recently developed to resolve this obstacle [87]. Researchers generated a zebrafish model expressing several human hematopoietic-specific cytokines (granulocyte-monocyte colony-stimulating factor, stem cell factor, and stromal cell-derived factor 1α) required for proper HSPC development and function. Xenotransplantation of primary human-derived HSPCs and primary human leukemia cells into these fish resulted in proper honing of HSPCs to their hematopoietic niches, enhanced proliferation, survival, multilineage differentiation, and self-renewal, providing an improved physiological, pre-clinical model for studying personalized therapies for leukemia and HSPC transplantation [87].

Monitoring the fate, behavior and responses of cancers at single-cell resolution provides one with information that cannot be obtained from studying cell populations in aggregate [88,89]. In the context of zebrafish xenografts, single-cell profiling has been employed in various ways to profile and understand cancer cell dynamics [29,82,84,90,91]. Toward improving treatments for colon cancer, single-cell measurements were used to distinguish differential sensitivity to therapeutics both within isogenic cancer cells and those derived from different patients [91]. In this respect, authors tested combination treatment regimens in colon cancer xenografts, and were able to delineate functional differences in proliferation, metastatic potential, and ability to induce neo-angiogenesis in zebrafish [91]. Single-cell monitoring has been employed in trying to understand both the behavior and responses of patient-derived rhabdomyosarcoma (RMS) cancer cells to targeted combination therapies [84]. Employing a FUCCI4 cell-cycle reporter, the responses of individual cells following combination treatments with the DNA-damaging agent, temozolomide and poly-ADP ribose polymerase (PARP) inhibitor, Olaparib were monitored to demonstrate combination efficacy in a prkdc^fb103/fb103^ il2rga^fb104/fb104^ Casper-strain zebrafish background. Photolineage tracing can be used to visualize the behavior of individual cells as tumor evolution and growth proceeds. To understand the developmental dynamics of cancer cells in RMS cells—a cancer characterized by the aberrant differentiation of muscle cells—a nuclear-localized, photo-convertible H2b-Dendra2 protein was used to monitor the fate of individual RMS cells belonging to phenotypically distinct subpopulations over the course of seven days. Using this approach, single cells could be traced, and different populations distinguished with dramatically different migratory and proliferative potential corresponding to their cell-of-origin maturation state [84].

Recently, exciting work has demonstrated the utility of the zebrafish xenograft model for visualizing in vivo responses to anti-cancer immunotherapy [29,92]. Zebrafish are used as avatars for anti-cancer immunotherapy to predict anti-tumor efficacy of chimeric antigen receptor T-cells (CAR-T cells) against patient-derived tumor cells, and to identify potential responders or non-responders to therapy. In order to visualize T-cell mediated killing of implanted patient-derived melanoma cells (hPMCs) in zebrafish, cancer cells were labeled with a live-cell fluorescing calcein dye. Upon T-cell mediated oncolysis, the dye is released to the extracellular space [29]. Tumor infiltrating lymphocytes (TILs) and peripheral bone marrow cells (PBMCs) were isolated from patients or healthy individuals respectively, and labeled with DiI, before injection into zebrafish, to visualize T-cell mediated killing by TILs, while PBMCs lacked any killing effect. Similarly, the ability of CAR- T cells to mediate cancer cell killing was visualized in vivo by co-injection of Raji lymphoma cells expressing CD19 and CD19-recognizing CAR-T cells. The killing effect of CAR-T cells, and TILs was also visualized in a metastatic melanoma model, where tumor cells were injected into the common cardinal vein (CCV) of zebrafish, followed by the tumor-targeting lymphocytes. Elimination of disseminated cancer melanoma cells were observed within 24 h post-injection [29]. Previous work by this group established a zebrafish model for replicating tumor-microenvironment interactions between cancer cells and stromal, cancer-associated fibroblasts (CAFs) [93], demonstrating that CAFs physically cluster with injected tumor cells, and can co-metastasize. Building on this system, the authors co-injected CAFs and hPMCs, and demonstrated that in both localized perivitelline injections, and CCV metastatic injections, CAFs protected hPMCs from T-cell mediated killing by CAR-T and TILs [29].

### 3.3. Limitations of the Zebrafish Xenograft Model for Cancer Drug Discovery

The zebrafish xenograft model is not without its limitations. Methodologically, the approach of drug dosing via immersion therapy in water comes with the corresponding challenges and considerations for translation to human biology. Technically, compounds with low water solubility will be inherently problematic for screening via immersion therapy, presenting some limitation for high-throughput screening approaches. Compounds with poor solubility can be directly injected into fish, or as noted in Section 3.1, adult fish can receive the drug by oral gavage [84], however these approaches are not necessarily amenable to high-throughput screening. Immersion therapy also differs from traditional drug administration techniques, as this approach exposes whole fish and the animal’s external tissues to drug, and this may affect both a compound’s efficacy against a given phenotype, or may induce different toxicities than would be observed with conventional dosing means.

Questions remain regarding the effective internal dose of a drug received in fish that are exposed via water immersion. Clinical decisions regarding therapeutic dosing windows typically rely on administration, distribution, metabolism, and excretion (ADME) measurements of a drug’s behavior in vivo using mammalian model systems. The development of approaches for the quantitation of drug ADME characteristics in zebrafish has been undertaken [84,94,95,96], and is expected to continue to grow, however more work needs to be done to reliably translate ADME findings for guiding clinical dosing.

The validity of cancer cells’ migration and invasion from the yolk sac into the bloodstream and other parts of the body as an active rather than passive process has been discussed [97] and the possibility has been raised that damaged blood vessels may permit cancer cell entry, or otherwise diffusion may spread cells throughout the yolk. Conversely, authors have observed cancer-cell specific migratory behavior, with different lines demonstrating different capacity to invade tissues [13,97], consistent with observed cell movement representing true invasion behavior. As a negative control, authors have suggested injection of fluorescent beads as a means to distinguish between passive and active movement from the yolk sac [97].

Beyond technical limitations, certain biological characteristics warrant consideration when leveraging zebrafish xenografts for drug discovery. Zebrafish have a greater capacity to regenerate multiple tissue types relative to humans and this fact needs to be considered when evaluating drug toxicity toward clinical translation [98]. Additionally, while zebrafish share a great degree of homology in disease-related genes, noteworthy differences in gene function highlight the need for some caution; for instance, the zebrafish mineralocorticoid receptor diverges in function from the human mineralocorticoid receptor in that the hormone progesterone acts as an agonist of the zebrafish receptor, but an antagonist of the human receptor [99].

### 3.4. Concluding Remarks

Our laboratories have been able to evaluate novel anti-cancer compounds utilizing zebrafish xenografts. We successfully tested a new class of compounds, Bcl-2 functional converters (BFCs) that convert Bcl-2 from a survival protein into a pro-apoptotic form [68,69], unpublished data. Cancer cells acquire resistance to chemotherapeutics, and resistance is in-part conferred by the upregulation of Bcl-2 protein expression, inhibiting apoptotic signals. Higher levels of Bcl-2 afford a therapeutic window whereby BFCs target and induce apoptosis in resistant cancer cells, without significant effect on normal tissue [68]. The use of zebrafish xenografts in these experiments provide valuable information on potential drug toxicity and effectiveness that will be used to set priority for further in vivo research and clinical translation. Additionally, we developed and demonstrated that a new nanoparticle light-delivery system was capable of therapeutic peptide delivery within an in vivo setting. The light-delivery system relied on 800 nm light to cause release of the peptides from nanoparticle bonds and endosomal entrapment [69]. While light-dependent release is straightforward in cell culture, fine tuning is needed to gain the same effects in vivo. Differences in power, duration and number of irradiations are impacted as animal tissue induces light scattering. Zebrafish provided an effective means to test and fine tune irradiating procedures that could release the drug in cancerous cells while not harming healthy animal tissue.

In conclusion, zebrafish xenograft models provide an effective platform for screening potential cancer drug candidates and evaluating cancer cell growth. Zebrafish offer a vertebrate anatomy with a hospitable in vivo environment containing relevant structures, i.e., extracellular matrix and flowing blood vessels, necessary for human tumor development. Zebrafish are relatively inexpensive in vivo models for early drug testing. They provide the means to test effectiveness and toxicity of potential drugs Furthermore, zebrafish provide the capability for high-throughput testing, in contrast to rodent models. As technology and methodology advance, zebrafish will become a vital part of cancer drug discovery by providing a bridge for translation of studies from in vitro to in vivo.

## Data Availability

Not applicable.
